# National Infant Immunization Week

**Published:** 2014-04-25

From April 26 through May 3, National Infant Immunization Week (NIIW) will focus attention on the role immunization plays in protecting infants from vaccine-preventable diseases. This year marks the 20th anniversary of both NIIW and the Vaccines for Children (VFC) program,* which provides vaccines at no cost for children who might otherwise not be vaccinated because of their caregiver’s inability to pay.

NIIW and VFC were initially created in response to a measles epidemic in which thousands of persons became infected as a result of low vaccination coverage among children aged <2 years (1). Since 1994, hundreds of communities across the country have joined to promote NIIW. Although immunization coverage among children has increased, recent outbreaks of measles and mumps in the United States underscore the importance of maintaining high immunization rates.

Throughout NIIW, local and state health departments, national immunization partners, and health-care professionals will conduct parent-focused events, clinician education activities, and other events to highlight the positive impact of vaccination on the lives of infants and to call attention to immunization achievements. To support these efforts, a variety of promotional and educational materials are available from CDC on the NIIW website (http://www.cdc.gov/vaccines/events/niiw/index.html).

NIIW is being observed simultaneously with World Immunization Week† (April 24–30), an initiative of the World Health Organization to promote and advance equity in the use of vaccines. Additionally, the winner of the annual CDC Childhood Immunization Champion Award, which recognizes local contributions to public health through work in childhood immunizations, will be announced.

## Footnotes

* Additional information available at http://www.cdc.gov/vaccines/programs/vfc/index.html.

† Additional information available at at http://www.who.int/campaigns/immunization-week/2014/en.
